# Thermal Stability and Inhibitory Action of Red Grape Skin Phytochemicals against Enzymes Associated with Metabolic Syndrome

**DOI:** 10.3390/antiox11010118

**Published:** 2022-01-05

**Authors:** Daniela Serea, Nina Nicoleta Condurache, Iuliana Aprodu, Oana Emilia Constantin, Gabriela-Elena Bahrim, Nicoleta Stănciuc, Silvius Stanciu, Gabriela Rapeanu

**Affiliations:** Faculty of Food Science and Engineering, Dunarea de Jos University of Galati, 111 Domneasca Street, 800201 Galati, Romania; daniela.serea@ugal.ro (D.S.); nina.condurache@ugal.ro (N.N.C.); Iuliana.Aprodu@ugal.ro (I.A.); oana.constantin@ugal.ro (O.E.C.); gabriela.bahrim@ugal.ro (G.-E.B.); Nicoleta.Stanciuc@ugal.ro (N.S.)

**Keywords:** red grape skins, anthocyanins, antioxidant activity, thermal stability, molecular modeling, biological activity

## Abstract

The present study focuses on heat-induced structural changes and the degradation kinetics of phytochemicals and antioxidant activity of red grape skin extract. The thermal degradation of anthocyanins, flavonoids, polyphenols, and antioxidant activity followed a first-order kinetic model, increasing with temperature due to the intensification of the degradation process. The activation energy (Ea) highlighted this phenomenon. Likewise, the kinetic and thermodynamic parameters certified the irreversible degradation of the bioactive compounds from the skin of the *Băbească neagră* grape variety. Both temperature and duration of heating had a significant impact on the content of bioactive compounds. In addition, the red grape skin extract inhibited certain enzymes such as α-amylase, α-glucosidase, lipase, and lipoxygenase, which are associated with metabolic syndrome and inflammation. Further knowledge on the possible inhibition mechanisms exerted by the major anthocyanins found in red grape skin extract on the metabolic syndrome-associated enzymes was gathered upon running molecular docking tests. Detailed analysis of the resulting molecular models revealed that malvidin 3-*O*-glucoside binds in the vicinity of the catalytic site of α-amylase and lipase, whereas no direct contact with catalytic amino acids was identified in the case of α-glucosidase and lipoxygenase.

## 1. Introduction

Wine production is one of the most important agricultural activities in the world and causes the generation of a large number of by-products, including skins, seeds, and stems. The management the by-products mentioned above presents serious environmental issues, as these residues have low pH, high content of organic matter, and can exert phytotoxic effects if applied to crops or wetlands [1].

The *Băbească neagră* variety (Engl. Black grandmother) represents an old native red grape variety cultivated in the southeastern part of Romania at the Dealu Bujorului vineyard. This grape variety is mainly used to produce light and fruity wines with 12–12.5% alcohol content [2]. Its skin is rich in bioactive compounds that have beneficial effects due to their antioxidant properties [3]. Red grape skins, as the main by-product of wineries, are rich in anthocyanins, flavonoids, and polyphenols [4,5]. 

Anthocyanins are water-soluble plant pigments responsible for the colors of flowers, fruits, vegetables, and grains that accumulate in the vacuoles of epidermal or sub-epidermal cells [6]. These compounds can prevent the appearance of several diseases, as they have properties such as anti-cardiovascular, anti-ulcer, anti-thrombotic, anti-inflammatory, anti-allergenic, and anti-coagulant. They are also known for their immunomodulatory, vasodilatory, and analgesic activities. There are also studies on their possible anticancer effect [6,7].

Heat treatment is often used in the food industry to ensure safety and preservation. It is known that heat treatment can lead to significant losses of biologically active compounds and, therefore, to decreases in antioxidant activity. The main factors that affect the anthocyanins’ stability depend on the processing and storage conditions [8]. Preventing anthocyanin degradation is an important issue that can benefit both processors and consumers [9].

Metabolic syndrome (MetS) represents a clinical syndrome characterized by multiple comorbidities such as hyperglycemia, abdominal obesity, arterial hypertension, and dyslipidemia. Together, these diseases can cause cardiovascular disease and early death [10], and have an impact on health care costs [11]. Pancreatic α-amylase is the key enzyme that decomposes dietary carbohydrates into simple monosaccharides in the digestive system. These are further degraded by α-glucosidases to glucose, which, on absorption, enters the bloodstream. Therefore, inhibiting α-amylase and α-glucosidase enzymes can suppress carbohydrate digestion, delay glucose uptake, and reduce blood sugar levels [12]. Pancreatic lipase is a key enzyme for the absorption of dietary triglycerides. Therefore, its inhibition affects fat hydrolysis and leads to a decrease in fat absorption [13].

The main purpose of this study is to investigate the kinetics of bioactive thermal degradation and their inhibitory activity against several metabolic enzymes. First, the extract from red grape skin was characterized in terms of total anthocyanin content (TAC), total flavonoid content (TFC), total polyphenol content (TPC), and antioxidant activity. Next, the anthocyanins from the extract were identified through HPLC. Further, the thermal degradation of phytochemicals and antioxidant activity from red grape skin extract was tested. The extract was heat-treated at temperatures ranging from 80 °C to 140 °C for various heating times (0–40 min). In addition, the extract was analyzed in terms of in vitro inhibitory capacity against the enzymes associated with diabetes, pancreatic disorders, and body inflammation. Thereby, the effect of the extract against α-amylase, α-glucosidase, lipase, and lipoxygenase (LOX) was tested and compared with the treatment drugs as positive standards. The single-molecule level interactions of the M3G with the metabolic syndrome-associated (MS) enzymes were finally explored upon running molecular docking tests in the attempt to explain the potential inhibitory activity.

## 2. Materials and Methods

### 2.1. Reagents

Ethanol 96%, Folin–Ciocalteau reagent (FC), DPPH (2,2-diphenyl-1-picrylhydrazyl), Trolox (6-hydroxy-2,5,7,8-tetramethylchromane-2-carboxylic acid), methanol (MeOH), potassium chloride solution (KCl), sodium acetate solution (CH_3_COONa), sodium carbonate (Na_2_CO_3_) 20%, sodium hydroxide (NaOH), aluminum chloride (AlCl_3_), α-glucosidase from Saccharomyces cerevisiae (≥10 units/mg protein), lipoxidase from Glycine max (soybean) (type I-B, 50,000 units/mg protein), pancreatic lipase (111.5 units/mg protein), α-amylase from porcine pancreas (type I-A, 700–1400 units/mg protein), phosphate buffer solution (PBS), linoleic acid (≥99%), p-nitrophenyl-α-D-glucopyranoside (≥97.5%), p-nitrophenyl palmitate (98%), Arabic gum, Triton X-100, starch solution, dinitrosalicylic acid (DNS), quercetin (≥95%), orlistat (≥98%), and acarbose (≥98%) were purchased from Sigma Aldrich (Germany). All chemicals and reagents used in the experiments were of analytical grade. The standards used for anthocyanins profile such as cyanidin, peonidin, malvidin, delphinidin 3-*O*-glucoside, cyaniding 3-*O*-glucoside, pelargonidin 3-*O*-glucoside, and malvidin 3-*O*-glucoside were obtained from Extrasynthese, GenayCedex (France, Genay).

### 2.2. Plant Material

Red grapes from the *Băbească neagră* variety were purchased from a local market in Galati, Romania (from the 2020 harvest). The grape skins were manually separated, washed with cold water, and rinsed with distilled water at a ratio of 1:2 (*w/w*). Then, they were wiped with paper towels to remove any residual pulp. The collected skins were freeze-dried using Alpha 1–4 LD plus equipment (CHRIST, Osterode am Harz, Germany) at −42 °C under a pressure of 10 Pa for 48 h. Finally, the dried skins were grounded and stored at 4 °C in a hermetically closed jar until further analysis.

### 2.3. Phytochemical Extraction

In brief, 1 g of red grape skin was mixed with 8 mL of ethanol 96% and 1 mL of 0.1 N HCl. The extraction was performed using a sonication water bath (MRC Scientific 193 Instruments, Holon, Israel) for 55 min at 50 °C, followed by centrifugation at 5000× *g* rpm for 10 min at 4 °C. The supernatant was collected and the extraction was repeated three times. Further, the collected supernatants were concentrated at 40 °C using a rotary evaporator under vacuum (AVC 2-18, Christ, Osterode, Germany). Finally, the extract was dissolved in ultrapure water (2 mg/mL) and phytochemically characterized.

### 2.4. Phytochemical Analysis of Biologically Active Compounds

The analysis of the bioactive compounds from the red grape skin extract was determined as previously described by Turturica et al. [14]. Thus, the TAC, TFC, TPC, and antioxidant activity of the extract were determined and calculated. TAC was expressed as mg cyanidin 3-*O*-glucoside/g dry weight (mg C3G/g dw). The TFC results were expressed as mg catechin equivalents/g dry weight (mg CE/g dw). The TPC was expressed as mg gallic acid equivalents/g dry weight (mg GAE/g dw), and the antioxidant activity as millimoles Trolox equivalents/g dry weight (mM TE/g dw). All bioactive compounds were measured using a Libra S22 UV-VIS spectrophotometer with data analysis software (Biochrom, Cambridge, UK).

### 2.5. Reverse-Phase HPLC Analysis of Anthocyanins

The anthocyanins identification from untreated skin extracts were determined using a procedure performed by Turturica et al. [14] using a Thermo Finnigan Surveyor HPLC system (Thermo Scientific, Waltham, MA, USA).

### 2.6. Heat Treatment

The heat treatment was performed in glass tubes with a screw cap on a digital heating block (Stuart SBH200D, Stafford, UK). An extract volume of 2 mL (2 mg/mL) with a pH = 3.2 was treated at temperatures between 80 and 140 °C. At regular time intervals (10, 20, 30, and 40 min), the samples were taken out of the bath and immediately placed in water with ice to prevent the bioactive compounds from further degrading. The phytochemical analysis was immediately performed as described above. The time and temperatures were chosen, taking into count the pasteurization, sterilization, and baking processes of various sweets, which need low temperatures, and longer time.

The HPLC profile of the thermally treated extract was also obtained as described above.

### 2.7. Kinetic Parameters

The bioactive’s content modification of the red grape skin was fitted to a first-order kinetic degradation model. The kinetic degradation rate (k), half-life of degradation (t_1/2_), decimal reduction time (D), and activation energy (Ea) were determined as described by Mercali et al. [15].

### 2.8. Thermodynamic Parameters

The thermodynamic parameters such as activation enthalpy (∆H), Gibbs free energy (∆G), and activation entropy (∆S) were estimated as described earlier by Vieira et al. [16].

### 2.9. In Vitro Enzymes Activity Inhibition

The inhibitory activity of red grape skin extract was tested against the chosen enzymes before and after the thermal treatment at 80 and 140 °C for 20 min.

#### 2.9.1. α-Amylase Inhibition Assay

Inhibition of α-amylase by red grape skin extract was assessed using the method described by Costamagna et al. [17]. Briefly, 100 μL of 0.5, 1 and 5 µg/mL extract (in ultrapure water) was mixed with 100 μL of α-amylase solution (1 mg/mL in 0.1 M PBS, pH = 6.9). Further, the samples were allowed to incubate at room temperature for 5 min. Next, 100 µL of 1% starch was added and incubated for 20 min at 37 °C. Then, 200 µL of DNS solution was added. The mixtures were thermally treated at 100 °C for 5 min using a thermostatic water bath (Digibath-2 BAD 4, Raypa Trade, Barcelona, Spain). The samples were diluted with 2 mL of distilled water and, finally, the absorbances were read at 540 nm using a UV-VIS spectrophotometer with data analysis software (Libra S22, Biochrom, Cambridge, UK). The standard inhibitor used as reference was acarbose. The inhibitory effect of the extract was expressed as an IC 50 value, which refers to the extract’s concentration that inhibits 50% of the enzyme.

#### 2.9.2. α-Glucosidase Inhibition Assay

The α-glucosidase inhibitory activity of the red grape skin extracts was also assessed according to Costamagna et al. [17]. In brief, 50 µL of α-glucosidase solution (1 mg/mL in 0.1 M PBS, pH = 6.9) and 50 µL of (0.5, 1, and 5 µg/mL) extract solutions were pre-incubated at room temperature for 5 min. Then, 50 µL of 25 mM p-nitrophenyl α-D-glucopyranoside and 1.6 mL of 0.1 M PBS (pH = 6.9) were added and the mixture was incubated for 15 min at 37 °C. Next, a volume of 800 µL of 0.2 M sodium carbonate was added and the absorbance was measured at 405 nm with a UV-VIS spectrophotometer (Libra S22, Biochrom, Cambridge, UK). The standard inhibitor used as reference was also acarbose. The inhibitory effect of the extract was expressed as IC50 (μg/mL).

#### 2.9.3. Lipase Inhibition Assay

For the lipase inhibitory effect of the extract, 50 μL of lipase solution (1 mg/mL in 0.1 M PBS, pH = 8.0) was mixed with 50 μL of red grape skin extract (0.5, 1, and 5 µg/mL) and incubated 5 min at room temperature. Next, 50 µL of the substrate (0.01 M p-nitrophenyl palmitate, Triton X-100, and Arabic gum) was added and incubated again at 37 °C for 20 min. Afterward, the samples were diluted with 1 mL of 0.1 M PBS at pH = 8.0, and the absorbance was read at 400 nm using a UV-VIS spectrophotometer with data analysis software (Libra S22, Biochrom, Cambridge, UK). The standard inhibitor used was orlistat, and the inhibitory effect was expressed as IC50 (μg/mL) [17].

#### 2.9.4. Lipoxygenase Inhibition Assay

For the LOX inhibition assay, 50 µL enzyme solution (1 mg/mL in 0.1 M PBS, pH = 9.0) was mixed with 50 µL of 0.5, 1, and 5 µg/mL extract solution and incubated for 5 min at room temperature. Afterward, 50 μL of the substrate (0.05 mM linoleic acid in 0.1 M PBS, pH = 9.0) was added and incubated for 20 min at 37 °C. Then, the samples were diluted in 2 mL of 0.1 M PBS (pH = 9.0) and absorbance was read in quartz cuvettes at 234 nm with a UV-VIS spectrophotometer with data analysis software (Libra S22, Biochrom, Cambridge, UK). The standard inhibitor used was quercetin and the inhibitory effect of the extract was expressed as the IC50 (μg/mL) [17].

### 2.10. Simulation of Anthocyanins Binding to the Enzymes by Molecular Docking

The molecular docking technique was used to check the potential sites on the surface of the enzymes related to the MS, which are directly involved in the interaction with M3G, the major anthocyanin found by means of HPLC in the extract of the red grape skin. The crystal structures of the MS-associated enzymes, namely α-amylase-pdb ID 6Z8L [18], α-glucosidase-pdb ID 5NN5 [19], lipase-pdb ID 1N8S [20], and lipoxygenase-pdb ID 3O8Y [21], were selected from the RCSB Protein Data Bank, whereas the model of the malvidin 3-*O*-glucoside was prepared using Hyperchem 8.0 software (Hypercube Inc., Gainesville, FL, USA). The refined models of the enzymes were used as receptors for the optimized model of the ligand (malvidin 3-*O*-glucoside) in the shape-complementarity-based rigid docking procedure, performed using the PatchDock algorithm [22]. The resulting protein-ligand models were ranked considering the interaction energy values and the top three models were analyzed in-depth for gathering advanced understanding on the possible mechanism involved in enzymes inhibition.

The interaction particularities of each enzyme-ligand complex were determining using dedicated tools such as PDBePISA [23,24] and VMD 1.9.3. [25].

### 2.11. Statistical Analysis

The analysis’ results were reported as mean ± standard deviation of triplicates. Statistical comparisons were made by a single analysis of variance (ANOVA). The Tukey method with a 95% confidence interval was employed for post hoc analysis; *p* < 0.05 was considered statistically significant. The statistical analysis was carried out using Minitab 18 software. The parameters of the kinetic models and the Arrhenius equation were estimated using linear regression.

## 3. Results and Discussion

### 3.1. The Phytochemical Content of Red Grape Skin Extract

The TAC of red grape skin extract was 23.20 ± 1.13 mg C3G/g dw. Sólyom et al. [26] reported a lower TAC of 3.81 ± 0.10 mg C3G/g dw in the red grape pomace, whereas Danisman et al. [27] identified a higher anthocyanin content of 60.70 ± 0.18 mg M3G/g dw in the grape juice. Constantin et al. [2] reported a lower TAC of 5.98 ± 0.08 mg C3G/g dw in the red grape skin of the *Babeasca neagra* variety.

In the red grape skin extract, a TFC content of 90.45 ± 11.67 mg CE/g dw was quantified, while Lavelli et al. [28] reported a TFC of 1.1 ± 0.1 mg CE/g dw.

The TPC of the red grape skin extract was 164.61 ± 44.15 mg GAE/g dw. Our results are higher than others who reported a TPC of 44.81 ± 10.48 mg GAE/g dw [26] in grape pomace and 43.9 ± 4.2 mg GAE/g dw [28] in the grape skin.

The inhibition of DPPH free radicals was measured to assay the antiradical activity of red grape skin extract. The extract showed a strong antiradical activity with a value of 1050.56 ± 308.97 mMol TE/g dw. A lower antioxidant activity value of 0.42 ± 0.1 mMol TE/g dw was reported by Sólyom et al. [26] for the grape pomace. Constantine et al. [2] reported an antioxidant activity content of 3.06 ± 0.04 µg TE/ mL^−1^ in the red grape skin of the *Babeasca neagra* variety.

The differences between our results and the one reported by other authors may be due to the plant matrix, different extraction conditions, grape variety, season, geographical origin, or the part of the grape used. However, the obtained data confirm that red grape skins represent a valuable source of biologically active compounds, especially anthocyanins, while also having high antioxidant activity.

### 3.2. HPLC Anthocyanins Profile Determination

**Figure 1:**
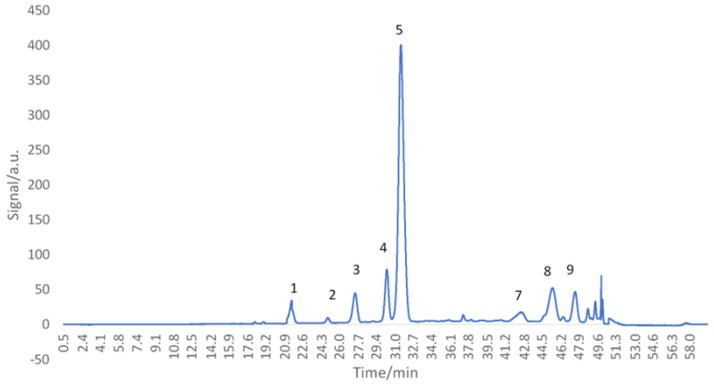
HPLC chromatograms of anthocyanin/anthocyanidin profile of *Băbească neagră* grape skin at 520 nm.

The anthocyanins chromatographic profile of untreated red grape skin extract was obtained (Figure 1). Nine anthocyanins were separated (Table 1): three anthocyanidins (cyanidin, peonidin, malvidin-peaks 6, 8, 9), five anthocyanidin-3-*O*-glucosides (delphinidin, cyanidin, petunidin, pelargonidin, malvidin-peaks 1, 2, 3, 4, 5), and one coumarylated anthocyanidin 3-*O*-glucosides (peonidin 3-*O*-coumaryl glucoside-peak 7). Similar profiles of anthocyanins were obtained for 14 grape varieties from the eastern Adriatic region; the anthocyanins identified were delphinidin, cyanidin, petunidin, peonidin, and malvidin, along with their 3-monoglucosides, acetylated, and p-coumarylated derivatives [29]. The main peak (5) was registered for the compound malvidin 3-*O*-glucoside, 13.83 ± 0.11 mg/g DW. Similar results were achieved for two black grapes, Gros noir and Muscat noir, in which malvidin 3-*O*-glucoside was the major anthocyanin component [30]. Kharadze et al. [31] obtained a similar profile for five grape varieties (Alexandrouli, Mujuretuli, Saperavi, Otskhanuri Sapere, Ojaleshi), with malvidin 3-*O*-glucoside as the predominant compound.

### 3.3. Thermal Degradation of Phytochemicals from Red Grape Skin

**Figure 2:**
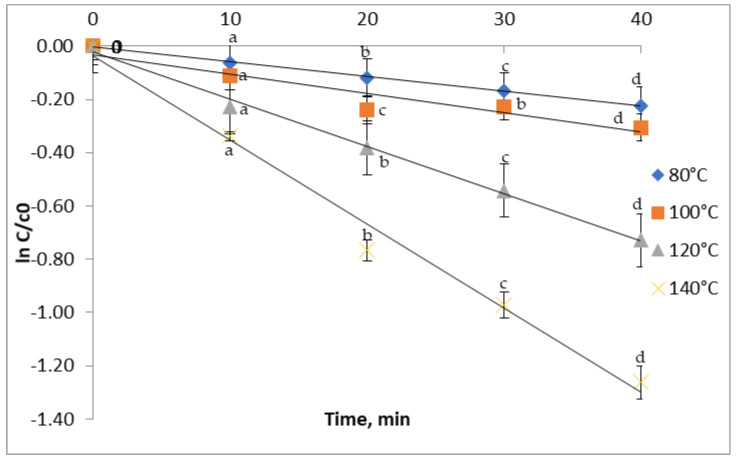
Isothermal degradation of TAC in the red grape skin extract treated at different temperatures.

For all studied temperatures, heating for 10 min led to significant losses of anthocyanins (Figure 2). Therefore, below 80 °C, after 10 min of treatment, a 6% reduction was observed, while at 140 °C, a 28.6% reduction was recorded. When increasing the heating time to 20 min, a more severe reduction, from 11.17% to 53.57% of the anthocyanins content, was registered at temperatures ranging from 80 °C to 140 °C. Similar results were obtained in studies performed on various plant matrices. Thus, Turturică et al. [32] reported the reduction of TAC by 47–63% after 60 min of heat treatment at temperatures between 90 °C and 120 °C. Karasu et al. [33] also reported a 20% decrease of the anthocyanin content of three grape varieties upon heat treatment for 8 h at 60 °C. When raising the temperature to 90 °C, they observed an 85% decrease in the anthocyanin content after heating at the same time.

**Figure 3:**
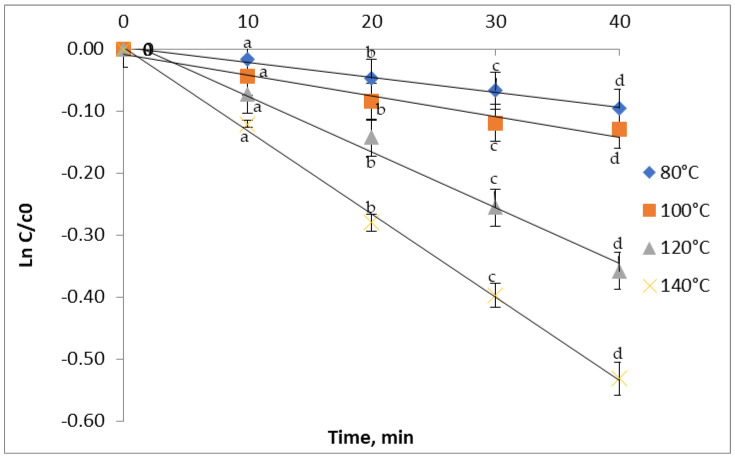
Isothermal degradation of TFC in red grape skin extract treated at different temperatures.

The changes in the TFC of red grape skin extracts after the treatment in the 80 to 140 °C temperature range as a function of heating time are presented in Figure 3. The flavonoid content follows a downward trend over the studied temperature range. Thus, by increasing the temperature from 100 to 140 °C for 40 min, a 12.18% to 41.25% reduction in the flavonoid content was observed. Our results are in agreement with Turturica et al. [14], who also reported a reduction of TFC from 36% to 67% in the plum extract (*Prunus domestica*) as a result of heat treatment between 70 °C and 110 °C for 15 min. To the contrary, Oancea et al. [34] reported an increasing trend of the TFC for the cherry extract. A temperature increase from 100 to 160 °C for 30 min caused a TFC increase from 153.06 ± 1.90 to 211.3 ± 9.52 mg CE/100 g dw.

**Figure 4:**
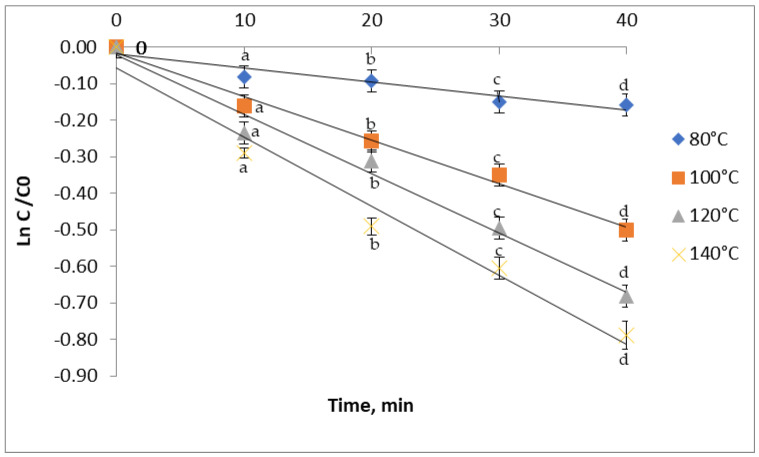
Isothermal degradation of TPC in red grape skin extract treated at different temperatures.

After 10 min of heating, the degradation of TPC begins and advances rapidly, with a significant decrease ranging from 7.8% at 80 °C to 23.5% at 140 °C (Figure 4). A reduction of 22.7% and 38.8% were observed as result of heating the samples at 100 and 120 °C, respectively, for 30 min. Similarly, the TPC from the plum extract reported by Turturica et al. [14] decreased from 4% to 23% in the temperature range of 70–90 °C and the degradation reached 43–72% at even higher temperatures of 100–110 °C.

**Figure 5:**
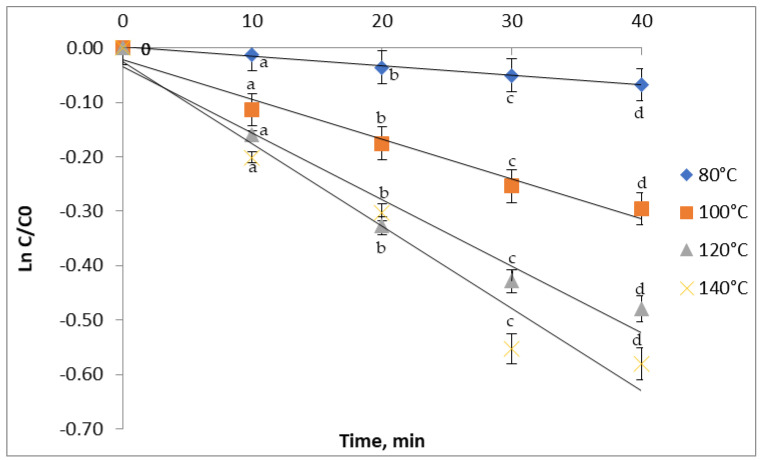
Isothermal degradation of the antioxidant activity in red grape skin extract treated at different temperatures.

The effect of heating on the antioxidant activity of the red grape skin extract is shown in Figure 5. The heat treatment caused the reduction of the antioxidant activity. In the 80–140 °C temperature range, a loss (1.21–18.25%) was observed after 10 min of heating, similar to the phytochemical content. The loss rate gradually increased with the heating time, and a gradual decrease in the antioxidant capacity was observed (Figure 5). Thus, after 40 min at 120 °C, a 38.05% decrease in the antioxidant capacity was noticed, with a 44.02% decrease after the treatment at 140 °C. This may be due to the phenolic compound’s degradation or other compounds responsible for the antioxidant activity of the extract during heating. These results are in agreement with other studies as Oancea et al. [34], who also found a decrease of 10–17% in the antioxidant activity of sour cherry skin extract after 60 min of thermal treatments at 100–150 °C.

**Figure 6:**
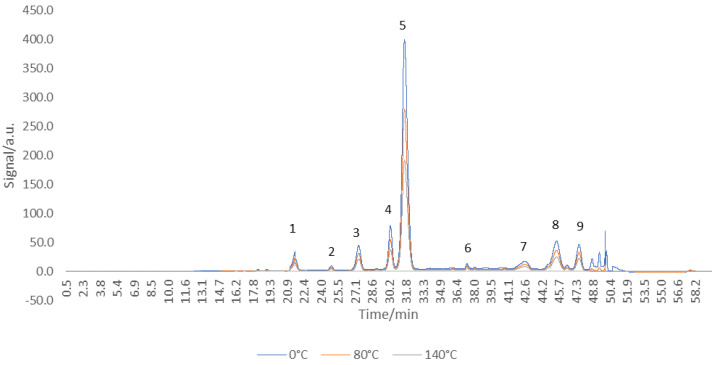
HPLC chromatograms of anthocyanin/anthocyanidin profile of *Băbească neagră* grape skin at 520 nm after thermal treatment at different temperatures for 20 min (Peak 1—delphinidin 3-*O*-glucoside; Peak 2—cyanidin 3-*O*-glucoside; Peak 3—petunidin 3-*O*-glucoside; Peak 4—pelargonidin 3-*O*-glucoside; Peak 5—malvidin 3-*O*-glucoside; Peak 6—cyaniding; Peak 7—peonidin 3-*O*-coumaryl glucoside; Peak 8—peonidin; Peak 9—malvidin).

Thermally treated red grape skin extract was analyzed using the HPLC technique. The extracts treated at 80 and 140 °C for 20 min were selected and analyzed in terms of the anthocyanins’ chromatographic profile (Figure 6) and the quantification of the main compound, malvidin 3-*O*-glucoside, was assessed.

All nine peaks initially identified were also obtained after the thermal treatment, with malvidin 3-*O*-glucoside still being the major one. When comparing the peaks, the susceptibility to degradation of anthocyanins is observed, the peaks intensity decreasing as the temperature increased. The malvidin 3-*O*-glucoside reduction was ~10% for 80 °C (12.34 ± 0.02 mg/g), and 32% for 140 °C (9.28 ± 0.04 mg/g). The chromatographic profile of the thermally treated extract confirms the results reported above, reinforcing the idea that the different temperature–time combinations cause a sequential reduction of anthocyanins from red grape skin extract.

### 3.4. Kinetics of Thermal Degradation

Kinetic models are being used for quick and economic assessment of food safety. Knowledge of degradation kinetics, including reaction order, rate constant, and activation energy, is of great importance in predicting food quality loss during heat treatment [30]. In our study, the obtained linear regression confirmed that the degradation of TAC, TFC, TPC, and antioxidant activity in red grape skin extract followed the first-order reaction. The degradation of phenolics was described in terms of degradation rate (k), the half-life of degradation (t_1/2_), decimal reduction time (D), and degradation energy of activation (Ea).

In this study, the anthocyanins extracted from red grape skin degraded at a lower rate, with k values ranging from 0.56 ± 0.01 × 10^−2^ min^−1^ at 80 °C to 3.16 ± 0.01 × 10^−2^ min^−1^ at a temperature of 140 °C (Table 2). For the thermal degradation of TFC, the k values increased from 0.24 ± 0.002 × 10^−2^ min^−1^ to 1.34 ± 0.01 × 10^−2^ min^−1^ with the temperature. For the TPC thermal degradation, the k value increased from 0.38 ± 0.002 × 10^−2^ min^−1^ to 1.89 ± 0.001 × 10^−2^ min^−1^. The increasing k values with the increasing temperature suggest a high thermal sensibility of the phytochemicals from the red grape skin. In the antioxidant activity case, there was a k value increase from 0.17 ± 0.03 × 10^−2^ min^−1^ to 1.51 ± 0.01 × 10^−2^ min^−1^. Similar to our study, Turturică et al. [32] reported k values increasing with the temperature from 70 °C to 120 °C for the anthocyanins’ thermal degradation in sweet cherry skin extract. Oancea et al. [34] also reported the increase of the TAC degradation rate with increasing temperature during the heat treatment of sour cherry skin extract. In the case of TFC, Turturica et al. [14] claimed that the kinetic velocity constants increased from 2.62 ± 0.24 × 10^−2^ min^−1^ to 9.02 ± 1.02 × 10^−2^ min^−1^ with increasing temperatures from 60 °C to 90 °C. For the TPC, an increase from 38.24 ± 10.83 × 10^−2^ min^−1^ at 60 °C to 45.63 ± 12.64 × 10^−2^ min^−1^ at 90 °C was recorded, suggesting a higher thermal sensitivity of polyphenols. The k value for the thermal degradation of the antioxidant activity reported by Turturica et al. [14] in the plum skin extract increased with increasing temperature. Higher k values were found in the sweet cherry skin extract, ranging from 9.27 ± 1.76 × 10^−2^ min^−1^ at 70 °C to 10.65 ± 2.83 × 10^−2^ min^−1^ at 110 °C [27].

As expected, the t_1/2_ and D values decreased with increasing temperature for all bioactive compounds analyzed (Table 2). The anthocyanins were the most susceptible to thermal degradation. The TFC had the highest half-life, especially at high heating temperatures. According to Turturică et al. [14], the half-life values of the TAC of the plum extract at temperatures between 70 °C and 110 °C were between 21.31 min and 11.00 min, respectively.

The resulting Ea values were 36.63 ± 0.07 kJ·mol^−1^ for TAC, 37.22 ± 0.02 kJ·mol^−1^ for TFC, 31.61 ± 0.01 kJ·mol^−1^ for TPC, and 43.59 ± 0.02 kJ·mol^−1^ for the antioxidant activity (Table 2). Higher Ea values suggest that a small temperature change is required to accelerate the degradation of the phytochemicals. Similar results were reported by Oancea et al. [34] for the cherry extract. Peron et al. [35] reported an Ea of 99.77 ± 0.87 kJ·mol^−1^ for the anthocyanin degradation in juçara extract (*Euterpe edulis Martius*) and 93.62 ± 0.44 kJ·mol^−1^ in “Italy” grape (*Vitis vinifera* L.) extract. Following the thermal degradation of anthocyanins in grape skin extract, Dyrby et al. [36] reported an Ea of 69.0 kJ·mol^−1^. Similar results of 35.50 ± 7.77 kJ mol^−1^ were also obtained by Turturica et al. [14] when applying thermal treatment to plum extract.

### 3.5. Thermodynamic Analysis of Antocyanins and Antioxidant Activity Degradation from Red Grape Skin Extract

When looking at the thermodynamic parameters regarding the anthocyanins from red grape skin, one can see that ΔH varied with the temperature in a narrow range of 33.69 ± 0.41 kJ/mol to 33.20 ± 0.25 kJ/mol (Table 3). Moreover, ΔH values were positive, revealing that the anthocyanins’ degradation was an endothermic reaction [37]. The fundamental criterion for the spontaneity of the chemical reaction is given by the constant ΔG, which in our study ranged from 120.01 ± 1.04 kJ/mol to 113.94 ± 1.04 kJ/mol. This indicates that the TAC’s degradation is non-spontaneous. The activation entropy (ΔS) represents the degree of disorder of molecules in a system and is related to the number of molecules with appropriate energy that can react [35]. The ΔS values determined for the reaction investigated in this study ranged from −193.50 ± 2.11 J/mol·K to −195.50 ± 2.09 J/mol·K. The negative values indicate that the structural freedom of the transition state is less than that corresponding to the reactants.

When studying the thermal degradation of anthocyanins from the sweet cherry skin extract, Turturica et al. [32] reported the increase of ΔG and ΔH values in the 70−120 °C temperature range. Mercali, et al. [37] reported enthalpy values of 69.24 kJ/mol to 69.49 kJ/mol for the purple potato. They argued that ΔG values, which ranged from 103.81 kJ/mol to 112.33 kJ/mol, indicate that the anthocyanin degradation is a non-spontaneous reaction. Sarkis et al. [38] studied the thermodynamic parameters of the anthocyanins from blackberry pulp, and reported ΔG values between 102.3–105.5 kJ/mol and ΔS values ranging from −114 to −144 J/mol·K.

The relatively low values of ΔH activation indicate that the formation of the activated complex is favored because the energy barrier to reach the transition state is low. As can be seen in Table 4, the activation enthalpy values were similar for all experimental conditions, ranging from 28.68 to 28.18 kJ/mol. The positive sign of ΔH means that the degradation process of the total polyphenol content is endothermic.

Turturica et al. [32] suggested that in the case of degradation of polyphenols in the extract obtained from cherries (*P. avium* L.), the values of ΔH increased from 2.68 to 3.26 kJ/mol, at a temperature of 323 K and 393 K, and ΔG recorded an increase from 86.26 kJ/mol at 323 K to 104.41 kJ/mol at a temperature of 393 K.

To assess whether the kinetic model provided in this study is thermodynamically possible, the thermodynamic parameters corresponding to flavonoid degradation were estimated. Table 5 shows the values of the activation enthalpy (ΔH), the free degradation energy (ΔG), and the activation entropy (ΔS) at different temperatures. From Table 5, it can be seen that ΔG ranged from 104.49 to 116.88 kJ/mol. ΔS is related to the number of molecules with adequate energy that can react effectively. The negative values of ΔS indicate a lower structural freedom than the reactants and also confirm that the process is irreversible; the values increased from −198.88 J/mol·K at 80 °C to −201.20 J/mol·K at 140 °C.

Turturica et al. [32] found, in the case of flavonoid degradation for the extract obtained from cherries (*P. avium* L.), increased values of ΔH from 2685.12 to 3.184 kJ/mol, at a temperature from 323 K to 393 K, respectively, and ΔG recorded an increase from 85.509 kJ/mol to 10.454 kJ/mol for the same temperature range.

The relatively low values of ΔH obtained for the antioxidant activity degradation (Table 6) indicate that the formation of the activated complex is favored because the energy barrier necessary to reach the transition state is low. As shown in Table 6, the activation enthalpy values were low for all experimental conditions, ranging from 40.66 ± 0.15 to 40.16 ± 0.17 kJ/mol. In general, ΔH is related to the strength of the bonds broken and formed in the transition state from the reactants, as well as to the solvation effect, which may differ for a given molecule. The ΔG values increased with the temperature, which also indicates a non-spontaneous reaction taking place. The negative ΔS values suggest that the heat-induced transition state has lower structural freedom than the reactants, while the thermal degradation process of the antioxidant activity is irreversible. 

When studying the thermodynamics of the antioxidant activity of the cherry extract, Turturica et al. [32] reported that the ΔH increased from 2.67 kJ/mol to 3.25 kJ/mol, and the ΔG registered a value of 10,405.71 kJ/mol in the 50–120 °C temperature range. Martynenko and Chen [9] reported ΔG values of 102.17–106.00 kJ/mol for the thermal degradation of the phytochemicals from purple potato extract.

### 3.6. The Inhibitory Effect of the Red Grape Skin Extract on the Activity of Metabolic Syndrome-Related Enzymes

Digestive enzymes are key enzymes that affect the digestion and absorption of carbohydrates in the diet. The decrease of the α-amylase and α-glucosidase activity directly leads to a slower decomposition of sucrose and starch into glucose, thereby inhibiting postprandial blood glucose [39].

The MS is a metabolic disorder with multiple etiologies, characterized by chronic hyperglycemia and disorders of carbohydrate and fat metabolism [17]. A therapeutic approach to lower postprandial hyperglycemia is to suppress glucose production and/or absorption from the gastrointestinal tract by inhibiting α-amylase or α-glucosidase enzymes [40]. The hypoglycemic agents used in clinical practice, such as acarbose, competitively inhibit α-glucosidase and α-amylase at the edge of the small intestine brush, which consequently delays carbohydrate hydrolysis and improves postprandial hyperglycemia [41].

LOX catalyzes the hydro-peroxidation of polyunsaturated fatty acids, which are precursors for the C6 aldehydes, hexanal, and hexenal [42]. The increasing levels of pancreatic lipase in the human body may lead to pancreatic disorders and obesity by catalyzing the hydrolysis of triglycerides into fatty acids [43].

The in vitro inhibitory effects of red grape skin extract were evaluated against α-glucosidase, α-amylase, pancreatic lipase, and LOX before and after the thermal treatment. The inhibitory capacity was tested using 0.5, 1, and 5 μg/mL extract concentrations, namely, and the results are presented in Table 7 and Table 8.

The untreated red grape skin extract presented high α-amylase inhibition at low concentrations. From Table 7, significant differences can be observed between the IC50 values of the extract and treatment drug (*p* < 0.05). Our results indicate that the untreated red grape skin extract can successfully replace the acarbose, with significantly lower extract concentrations necessary to inhibit 50% of the α-amylase (*p* < 0.05). The same behavior can be observed for the α-glucosidase inhibition, with the IC 50 values being 1.06 ± 0.16 µg/mL for the extract and 1.75 ± 0.14 µg/mL for acarbose. In contrast to our results, Lavelli et al. [44] reported higher α-glucosidase inhibition activity of two varieties of grape skin extracts, with the IC 50 value in the range of 30.9 ± 1.5 µg GAE/mL to 93.1 ± 3.2 μg GAE/ mL, and for amylase, in the 12.5 ± 0.8–26.3 ± 0.2 μg GAE/mL range.

The extract obtained from the skin of red grapes also showed inhibitory activity against LOX (Table 7). However, the IC50 value of the extract against LOX (1.64 ± 0.07 μg/mL) was significantly higher than for quercetin (1.18 ± 0.02 μg/mL).

The red grape skin extract exhibited dose-dependent inhibition against pancreatic lipase activity in the in vitro test system. The inhibitory activity of the extract on pancreatic lipase increased with the increasing concentration of the extract. The IC50 value of the red grape skin extract was 7.62 ± 0.86 μg/mL, whereas for orlistat it was significantly lower (*p* < 0.05), with a value of 3.18 ± 0.33 μg/mL. However, You et al. [45] evaluated the effects of Muscadine grape skin extract against the pancreatic lipase and they reported a higher IC50 value (16.90 ± 0.07 µg/mL).

The enzymes’ inhibitory activity was also tested for extract thermally treated at 80 °C and 140 °C for 20 min; the results are presented in Table 8.

For each inhibitory activity tested, the IC50 values significantly increased with increasing temperature (*p* < 0.05) (Table 8). Thus, for α-amylase the IC50 value increased from 3.06 ± 0.30 to 5.06 ± 0.31 µg/mL extract. A smaller increase of IC50 value was observed in the case of α-glucosidase from 1.06 ± 0.16 to 1.79 ± 0.02 µg/mL extract. The lipase inhibitory activity presented the highest increase from 7.62 ± 0.86 to 13.37 ± 1.71 µg/mL extract. The LOX inhibitory activity presented an increase from 1.64 ± 0.71 to 2.75 ± 0.01 µg/mL extract. The increase in the IC50 value means that the polyphenolic compounds from the red grape skin extract are degraded, as stated earlier, and a higher concentration is necessary to inhibit 50% of the enzyme.

**Figure 7:**
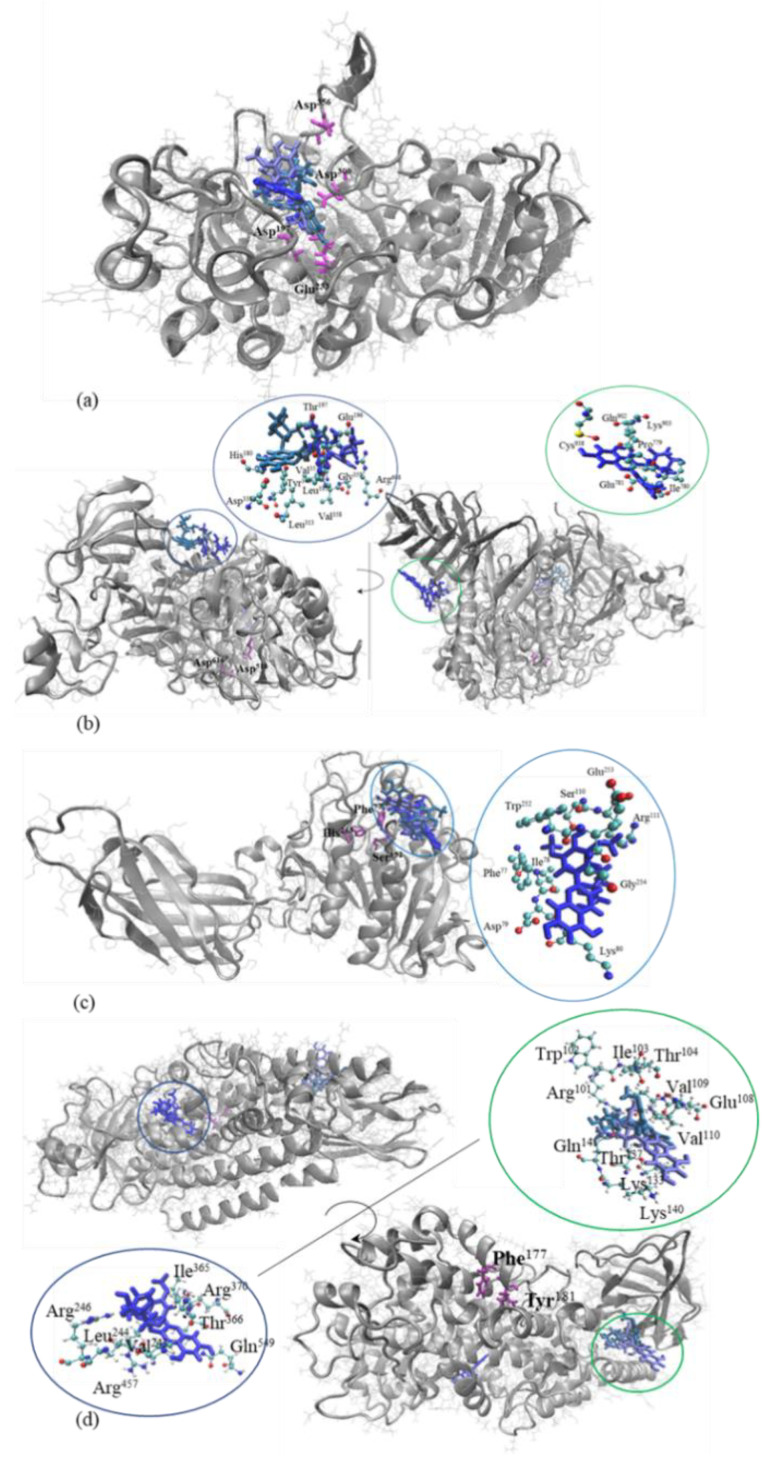
Superposition of the top three scoring models resulting from the molecular docking tests, showing the complexes formed by α-amylase (**a**), α-glucosidase (**b**), lipase (**c**), and lipoxygenase (**d**) represented in silver, with M3G is represented in different shades of blue in licorice style. The catalytic amino acids are highlighted in purple. The insets present details on the ligand binding sites. Images were prepared using VMD software.

Further atomic level details on the interaction between the metabolic syndrome-associated enzymes and the major anthocyanin found in the in the extract of the red grape skin were collected upon running in silico docking tests. The complexes resulting upon M3G binding to the α-amylase, α-glucosidase, lipase, and lipoxygenase are presented in Figure 7.

A close analysis of the docking models involving α-amylase as receptor indicate that, in the cases of all three top scoring complexes, the M3G attached to the enzyme surface in the close vicinity of the catalytic site (Figure 7a). The anthocyanin established contacts with Trp^58^, Trp^59^, Tyr^62^, Leu^162^, Thr^163^, Leu^165^, Asp^300^, His^305^, and Gly^306^. Our observations are in good agreement with Wu et al. [46], who investigated the inhibitory mechanism exhibited by the gallocatechin gallate on α-amylase and reported the same binding site. As in the study of Wu et al. [46], the binding site of the M3G molecule partially overlapped the active site of α-amylase, interacting with Asp^300^, which is a pivotal amino acid involved in exerting catalytic activity [18]. This observation suggests that ligand binding to the enzyme surface might interfere with the catalytic activity of α-amylase, therefore supporting the inhibitory potential measured experimentally.

In the case of α-glucosidase, it appears that none of the top three scoring models involves the direct contact of M3G molecules with catalytic amino acids. According to Roig-Zomboni et al. [19], the main active site of α-glucosidase is located on a narrow pocket at the C-terminal ends of the β-strands found in the catalytic GH31 domain, with the catalytic nucleophile and acid/base activity being attributed to Asp^518^ and Asp^616^, respectively. As shown in Figure 7b, one potential ligand biding site is located in a wide-open cavity on the proteins surface, defined by amino acids belonging to the proximal β-sheet domain (Pro^779^, Ile^780^, and Glu^781^) and the proximal β-sheet domain (Gln^902^, Lys^903^, and Cys^938^). A second binding site of M3G molecule, able to accommodate with rather high affinity the ligand in two different relative positions, is located in the N-terminal proximal β-sheet domain (Figure 7b). The amino acids that establish direct contacts with the ligand in these two cases are His^180^, Val^193^, Leu^195^, Glu^196^, Thr^197^, Leu^313^, Asp^338^, Tyr^340^, and Val^357^ [21].

The results of the docking simulations involving the lipase as receptor indicated that M3G molecules attach with high affinity to the shallow cavity located on the surface of the enzyme, in the close vicinity of Phe^77^, one amino acid of the catalytic triad [43]. No important differences among the three top scoring complexes were found in terms of the binding site (Figure 7c). The M3G binding in the neighborhood of the catalytic site might interfere with substrate recognition and its transformation by lipase. These observations are in good agreement with the findings of Liu et al. [47], who used the molecular docking simulations to gather insights into the inhibitory mechanism exerted by different bioactive compounds from Ginkgo biloba on the activity of pancreatic lipase. They reported that isoginkgetin, bilobetin, and ginkgetin preferentially attached to the catalytic cavity of lipase, establishing contacts with Phe^77^, His^263^, and Ser^152^.

Two different binding sites of M3G to lipoxygenase were identified when checking the complexes with the highest interaction energy values resulting from the molecule docking tests (Figure 7c). Both potential ligand-binding sites located in narrow pockets on the protein surface are positively charged. The first binding site is rather polar and involves three Arg residues located in positions 246, 370, and 457. The second binding site consists mainly of nonpolar residues and contains three positively charged amino acids (Arg^101^, Lys^133^, and Lys^140^) and one negatively charged amino acid (Glu^108^). Both ligand-binding sites are located far from Phe^177^ and Tyr^181^ residues, which play a crucial role in ensuring access to catalytic iron [21]. Our observations comply with previous findings of Mahesha, et al. [48], who studied the inhibitory effects of soy isoflavones and showed that the inhibitors attach to the enzyme in a non-competitive manner, at a different site in respect to the substrate. Moreover, they indicated a dual mechanism for LOX inhibition by the bioactives; they are able to scavenge the free radical providing an electron to the Fe^3+^ of the enzyme, which is converted to the resting state and prevents further enzyme activation by competing with the hydroperoxide [48]. Due to their structural particularities, polyphenols in general are able to chelate with iron and alter the redox chemistry, causing the inactivation of LOX [48].

Although the ligand does not bind in the vicinity of the active site of the enzymes such as to compete with the substrate, due to the intrinsic molecular instability [21], some local rearrangements disturbing the conformation of the active site might appear.

A wide range of bioactive compounds are found in red grape skin extract and even though their concentration is rather low compared to M3G, they may impact the activity of the metabolic syndrome-associated enzymes. Therefore, the cumulative contribution of the bioactive compounds should be considered when trying to explain the experimental results.

## 4. Conclusions

In conclusion, the present study investigated the effect of heat treatment on phenolic compounds from red grape skin. The effect of high temperatures on the phytochemical degradation from red grape skin extracts was examined based on a detailed kinetic study. The degradation of TAC, TFC, TPC, and antioxidant activity during isothermal treatments followed the first-order kinetic model. The kinetic and thermodynamic parameters showed a high-temperature dependence for the phytochemicals and moderate temperature dependence for the antioxidant activity from the red grape skin. In addition, the extract presented in vitro inhibitory activity against α-amylase, α-glucosidase, lipase, and lipoxygenase. This fact suggests that red grape skin extract might be valuable for altering the activity of the metabolic syndrome-associated enzymes. Further tests involving biological assays are needed to confirm the possibility of using red grape skin extract as a natural treatment for reducing the occurrence of MS. The information provided by this study may be useful for optimizing food industrial processing conditions, in order to minimize biologically active compound losses. Based on these findings, further studies are being developed by our research team for the efficient utilization of these pigments in actual food products and/or nutraceuticals.

## Figures and Tables

**Table 1:** The compounds detected in untreated extracts of grape skins by HPLC.

Peak	Compound	*t*_r_, min	Anthocyanins, mg/g
1	Delphinidin 3-*O*-Glucoside	21.02 ± 0.02	1.25 ± 0.02
2	Cyanidin 3-*O*-Glucoside	25.01 ± 0.01	0.58 ± 0.00
3	Petunidin 3-*O*-Glucoside	27 ± 0.03	NQ
4	Pelargonidin 3-*O*-Glucoside	29.9 ± 0.01	2.91 ± 0.01
5	Malvidin 3-*O*-Glucoside	30.9 ± 0.02	13.83 ± 0.11
6	Cyanidin	37.8 ± 0.01	0.61 ± 0.00
7	Peonidin 3-*O*-Coumaryl Glucoside	42.2 ± 0.02	NQ
8	Peonidin	45.1 ± 0.04	2.26 ± 0.03
9	Malvidin	47.1 ± 0.04	2.14 ± 0.01

NQ—Not Quantified.

**Table 2:** The kinetic parameters of thermal degradation of phytochemicals from red grape skin extract.

Compound	T (°C)	K × 10^−2^ (min^−1^)	t_1/2_ (min)	D (min)	Ea (kJ·mol^−1^)
TAC	80	0.56 ± 0.01 ^d^	123.77 ± 0.21 ^a^	411.17 ± 4.54 ^a^	36.63 ± 0.07 ^a^
	100	0.72 ± 0.01 ^c^	96.27 ± 0.11 ^b^	319.80 ± 0.51 ^b^	
	120	1.77 ± 0.48 ^b^	39.16 ± 0.01 ^c^	130.08 ±1.01 ^c^	
	140	3.16 ± 0.01 ^a^	21.93 ± 0.30 ^d^	72.86 ± 0.81 ^d^	
TFC	80	0.24 ± 0.002 ^d^	288.81 ± 0.40 ^a^	959.41 ± 3.71 ^a^	37.22 ± 0.02 ^a^
	100	0.33 ± 0.01 ^c^	210.04 ± 0.93 ^b^	697.75 ± 1.71 ^b^	
	120	0.90 ± 0.003 ^b^	77.01 ± 0.91 ^c^	255.84 ± 1.71 ^c^	
	140	1.34 ± 0.05 ^a^	51.72 ± 0.91 ^d^	171.83 ± 0.30 ^d^	
TPC	80	0.38 ± 0.002 ^d^	182.40 ± 6.04 ^a^	605.94 ± 0.20 ^a^	31.61 ± 0.07 ^a^
	100	1.19 ± 0.01 ^c^	58.24 ± 3.01 ^b^	193.49 ± 0.51 ^b^	
	120	1.62 ± 0.003 ^b^	42.78 ± 1.01 ^c^	142.13 ± 0.91 ^c^	
	140	1.89 ± 0.001 ^a^	36.67 ± 3.03 ^d^	121.82 ± 0.41 ^d^	
Antioxidant Activity	80	0.17 ± 0.03 ^d^	407.73 ± 1.20 ^a^	1354.46 ± 3.01 ^a^	43.59 ± 0.02 ^a^
	100	0.73 ± 0.01 ^c^	94.95 ± 0.26 ^b^	315.42 ± 0.71 ^b^	
	120	1.23 ± 0.01 ^b^	56.35 ± 0.20 ^c^	187.20 ± 0.91 ^c^	
	140	1.51 ± 0.01 ^a^	45.90 ± 1.12 ^d^	152.48 ± 0.02 ^d^	

Measurements are expressed as mean ± SD of triplicates. Within a set of experiment, means that on the same column do not share a letter (^a–d^) are significantly different at *p* < 0.05.

**Table 3:** Thermodynamic parameters obtained for TAC degradation in red grape skin extract.

Temperature (°C)	ΔH (kJ/mol)	ΔS (J/mol·K)	ΔG (kJ/mol)
80	33.69 ± 0.41 ^a^	−193.50 ± 2.11 ^a^	120.01 ± 1.04 ^a^
100	33.53 ± 0.25 ^a^	−197.44 ± 2.12 ^c^	107.17 ± 1.06 ^d^
120	33.36 ± 0.17 ^a^	−195.39 ± 3.06 ^b^	110.15 ± 1.08 ^c^
140	33.20 ± 0.25 ^a^	−195.50 ± 2.09 ^b^	113.94 ± 1.04 ^b^

Measurements are expressed as mean ± SD of triplicates. Values (mean ± SD) from a column that shares the same letter are not significantly different (*p* > 0.05).

**Table 4:** Thermodynamic parameters obtained for TPC degradation in red grape skin extract.

Temperature (°C)	ΔH (kJ/mol)	ΔS (J/mol·K)	ΔG (kJ/mol)
80	28.68 ± 0.14 ^a^	−210.94 ± 0.54 ^c^	103.14 ± 1.00 ^d^
100	28.51 ± 0.17 ^a^	−206.71 ± 0.21 ^a^	105.62 ± 1.12 ^c^
120	28.34 ± 0.09 ^a^	−208.90 ± 0.14 ^b^	110.44 ± 1.16 ^b^
140	28.18 ± 0.14 ^a^	−211.92 ± 0.14 ^c^	115.70 ± 1.18 ^a^

Measurements are expressed as mean ± SD of triplicates. Values (mean ± SD) from a column that shares the same letter are not significantly different (*p* > 0.05).

**Table 5:** Thermodynamic parameters obtained for TFC degradation in red grape skin extract.

Temperature (°C)	ΔH (kJ/mol)	ΔS (J/mol·K)	ΔG (kJ/mol)
80	34.28 ± 0.21 ^a^	−198.88 ± 0.79 ^a^	104.49 ± 1.04 ^d^
100	34.12 ± 0.14 ^a^	−202.34 ± 0.80 ^c^	109.59 ± 1.06 ^c^
120	33.95 ± 0.20 ^a^	−199.51 ± 0.95 ^ab^	112.36 ± 1.12 ^b^
140	33.79 ± 0.14 ^a^	−201.20 ± 0.79 ^bc^	116.88 ± 1.14 ^a^

Measurements are expressed as mean ± SD of triplicates. Values (mean ± SD) from a column that shares the same letter are not significantly different (*p* > 0.05).

**Table 6:** Thermodynamic parameters obtained for antioxidant activity degradation in red grape skin extract.

Temperature (°C)	ΔH (kJ/mol)	ΔS (J/mol·K)	ΔG (kJ/mol)
80	40.66 ± 0.15 ^a^	−183.68 ± 2.08 ^c^	105.50 ± 1.04 ^d^
100	40.49 ± 0.52 ^a^	−178.65 ± 2.14 ^a^	107.13 ± 1.07 ^c^
120	40.33 ± 1.02 ^a^	−180.69 ± 2.02 ^b^	111.34 ± 1.09 ^b^
140	40.16 ± 0.17 ^a^	−184.77 ± 2.07 ^c^	116.47 ± 1.04 ^a^

Measurements are expressed as mean ± SD of triplicates. Values (mean ± SD) from a column that shares the same letter are not significantly different (*p* > 0.05).

**Table 7:** The inhibitory capacity (IC50 values; μg/mL) of the red grape skin extract on α-amylase, α-glucosidase, lipase, and lipoxygenase (LOX) activity before thermal treatment.

Sample	IC50 (μg/mL Extract)			
	α-Amylase	α-Glucosidase	Lipase	LOX
Extract	3.06 ± 0.30 ^a^	1.06 ± 0.16 ^a^	7.62 ± 0.86 ^b^	1.64 ± 0.71 ^a^
Acarbose	3.91 ± 0.44 ^b^	1.75 ± 0.14 ^b^	-	-
Orlistat	-	-	3.18 ± 0.33 ^a^	-
Quercetin	-	-	-	1.18 ± 0.20 ^b^

Values from a column that share a letter are not significantly different (*p* > 0.05). Measurements are expressed as mean ± SD of triplicates.

**Table 8:** The inhibitory capacity (IC50 values; μg/mL) of the red grape skin extract on α-amylase, α-glucosidase, lipase, and lipoxygenase (LOX) activity thermally treated at 80 °C and 140 °C.

Temperature	IC50 (μg/mL Extract)			
	α-Amylase	α-Glucosidase	Lipase	LOX
25 °C	3.06 ± 0.30 ^a^	1.06 ± 0.16 ^a^	7.62 ± 0.86 ^a^	1.64 ± 0.71 ^a^
80 °C	3.14 ± 0.33 ^a^	1.02 ± 0.17 ^a^	8.70 ± 0.38 ^b^	2.29 ± 0.01 ^b^
140 °C	5.06 ± 0.31 ^b^	1.79 ± 0.02 ^b^	13.37 ± 1.71 ^c^	2.75 ± 0.01 ^c^

Values from a column that share a letter are not significantly different (*p* > 0.05). Measurements are expressed as mean ± SD of triplicates.

## Data Availability

The data that support the findings of this study are available from the corresponding author, G.R., upon reasonable request. The data are not publicly available due to privacy.
